# Editorial. In this issue

**DOI:** 10.1590/S1677-5538.2016.01.01

**Published:** 2016

**Authors:** Stênio de Cássio Zequi

**Affiliations:** Divisão de Urologia do A.C. Camargo Cancer Center. Fundação A. Prudente, São Paulo, Brasil

The best treatment for small renal masses has been subject of debates. Despite of the small size, endophitic lesions may represent a challenge, especially in posterior side of the kidneys. In the section Difference of Opinion (pages 3-10), experts of Interventional radiology of Albert Einstein Hospital in São Paulo, claims for cryoablation, while the colleagues from Sonora University, Mexico and Southern California University, are favorable to robotic partial nephrectomy.

At the Review Article, an interesting multidisciplinary study, performed by Tiseo et al., from the Urology and Rheumatology groups from the University of São Paulo, is presented. The authors reviewed extensively the literature since 1970, regarding the influence of the more prevalent rheumatologic diseases and/or treatments on male fertility. These are useful information for the urologist’s daily practice, which might include these investigations during the initial anamnesis of infertile men. Among urologists, it is almost consensual to avoid brachytherapy in patients previously submitted to transurethral resection of the prostate (TURP); two Spanish groups from Cantabria reported good oncological and functional outcomes (only 1.7% of urinary incontinence) in 57 patients submitted to dose brachytherapy implantation after TURP, in a medium follow up of 104 months (page 47). Middle East Groups have identified prognostic factors for urinary sepsis after transrectal prostatic biopsy in Lebanon (page 60), and a differential expression of leucocytes and of neutrophil to lymphocyte ratio in Turkish patients with localized testis cancer (page 53).

Moving to minimally invasive surgery, an electronic survey revealed that almost half of Portuguese surgeons are favorable to transvaginal extraction of kidney, after laparoscopic nephrectomies; interestingly, female surgeon gynecologists preferred the vaginal approach (page 78).

A Brazilian group (Marchini et al., page 90), reported the feasibility, the pitfalls and initial results of the laparoscopic single port bilateral nephrectomy in porcine models, performed by post-graduated students.

On page 154, Parente et al. proposed the use of a high pressure balloon to investigate the etiology of ureteropelvic junction obstruction (UPJO) in children: when at fluoroscopy a “waist” in the balloon is verified, an intrinsic UPJO is considered, and is managed by a dismembered pyeloplasty; on the other hand, when there is “no waist” at fluoroscopy, only a vascular hitch is performed regarding the lower pole crossing vessels.

In relation to ischemic priapism (IP), according to Ufuk et al., men with IP present higher mean platelet volumes than healthy controls; probably these elevated platelets volumes contribute in the venno-occlusive mechanisms. In an induced IP rat model, the peritoneal infusion of dipyridamole was tested as protective drug against the endothelial reperfusion injury processes (page 118).

A new method of semen microcentrifugation, developed by Hallak’s group is proposed as an easy, low cost, and reproducible option for the investigation of non-obstructive azospermia. Sperm positivity was identified in 21 of 148 samples (page 132).

*Stênio de Cássio Zequi* Editor Associado, International Braz J Urol Divisão de Urologia do A.C. Camargo Cancer Center Fundação A. Prudente, São Paulo, Brasil

